# Image similarity evaluation of the bulk-density-assigned synthetic CT derived from MRI of intracranial regions for radiation treatment

**DOI:** 10.1371/journal.pone.0185082

**Published:** 2017-09-19

**Authors:** Shin-Wook Kim, Hun-Joo Shin, Jin-Ho Hwang, Jin-Sol Shin, Sung-Kwang Park, Jin-Young Kim, Ki-Jun Kim, Chul-Seung Kay, Young-Nam Kang

**Affiliations:** 1 Department of Radiation Oncology, Incheon St. Mary’s Hospital, College of Medicine, The Catholic University of Korea, Seoul, Korea; 2 Department of Biomedical Engineering, College of Medicine, The Catholic University of Korea, Seoul, Korea; 3 Department of Radiation Oncology, Busan Paik Hospital, Inje University, Busan, Korea; 4 Department of Radiation Oncology, Haeundae Paik Hospital, Inje University, Busan, Korea; 5 Department of Radiology, Incheon St. Mary’s Hospital, College of Medicine, The Catholic University of Korea, Seoul, Korea; 6 Department of Radiation Oncology, Seoul St. Mary’s Hospital, College of Medicine, The Catholic University of Korea, Seoul, Korea; North Shore Long Island Jewish Health System, UNITED STATES

## Abstract

**Objective:**

Various methods for radiation-dose calculation have been investigated over previous decades, focusing on the use of magnetic resonance imaging (MRI) only. The bulk-density-assignment method based on manual segmentation has exhibited promising results compared to dose-calculation with computed tomography (CT). However, this method cannot be easily implemented in clinical practice due to its time-consuming nature. Therefore, we investigated an automatic anatomy segmentation method with the intention of providing the proper methodology to evaluate synthetic CT images for a radiation-dose calculation based on MR images.

**Methods:**

CT images of 20 brain cancer patients were selected, and their MR images including T1-weighted, T2-weighted, and PETRA were retrospectively collected. Eight anatomies of the patients, such as the body, air, eyeball, lens, cavity, ventricle, brainstem, and bone, were segmented for bulk-density-assigned CT image (_B_CT) generation. In addition, water-equivalent CT images (_W_CT) with only two anatomies—body and air—were generated for a comparison with _B_CT. Histogram comparison and gamma analysis were performed by comparison with the original CT images, after the evaluation of automatic segmentation performance with the dice similarity coefficient (DSC), false negative dice (FND) coefficient, and false positive dice (FPD) coefficient.

**Results:**

The highest DSC value was 99.34 for air segmentation, and the lowest DSC value was 73.50 for bone segmentation. For lens segmentation, relatively high FND and FPD values were measured. The cavity and bone were measured as over-segmented anatomies having higher FPD values than FND. The measured histogram comparison results of _B_CT were better than those of _W_CT in all cases. In gamma analysis, the averaged improvement of _B_CT compared to _W_CT was measured. All the measured results of _B_CT were better than those of _W_CT. Therefore, the results of this study show that the introduced methods, such as histogram comparison and gamma analysis, are valid for the evaluation of the synthetic CT generation from MR images.

**Conclusions:**

The image similarity results showed that _B_CT has superior results compared to _W_CT for all measurements performed in this study. Consequently, more accurate radiation treatment for the intracranial regions can be expected when the proper image similarity evaluation introduced in this study is performed.

## Introduction

Owing to its direct connection with the electron density, computed tomography (CT) is the standard for the current radiation treatment planning (RTP) methodology [1]. CT has excellent geometrical accuracy and enables accurate radiation-dose calculations. However, in the intracranial regions, accurate delineation of the target volumes when using only CT images is impractical because of the poor soft-tissue contrast of these images [2]. Therefore, multimodal clinical image acquisition techniques, such as magnetic resonance imaging (MRI) and positron emission tomography (PET), are additionally utilized for the accurate delineation of the target volumes [3–5]. At present, image registration between MRI or PET and CT is the standard for the radiation treatment of conditions such as brain tumors, prostate cancer, and spine tumors [6, 7]. However, some authors have reported that an inevitable uncertainty arises systematically due to the essential image registration procedure [8–10]. To overcome this problematic uncertainty, several methods for radiation-dose calculation have been investigated over previous decades, focusing on the sole use of MRI in RTP systems. These are mainly categorized into two approaches—CT-dependent [11–14] and CT-independent [15–18]. For example, CT-dependent approaches involve image registration procedures between MRI and CT. Then, averaged CT atlases are matched to a new MRI for a radiation-dose calculation. The limitation is that this approach is literally dependent on CT and may not be able to reduce the registration uncertainty satisfactorily. Moreover, CT-dependent approaches are considered insensitive to patients’ abnormal anatomies. On the contrary, CT-independent approaches involve assigning electron density information to a region on the MRI. These approaches could be classified into water-equivalent density assignment and bulk-density assignment. Water-equivalent density assignment does not involve inhomogeneity correction, whereas bulk-density assignment involves density assignment of a few atlases on MR images. Some researchers have investigated water-equivalent density assignment as a radiation-dose calculation method for MRI use only. The authors calculated radiation doses in a water-equivalent patient-shaped geometry, and the results indicated a calculated dose difference of up to 2.5% compared to the dose calculation based on CT [19]. Other researchers have investigated bulk-density assignment as a radiation-dose calculation method for MRI use only. They calculated radiation doses using a bulk-density assigned to a manually defined anatomy such as air, bone, fat, or soft tissue. This method has exhibited promising results, in that the calculated dose difference extends up to only 1% compared to the dose calculation based on CT [20]. However, the bulk-density assignment method cannot be easily implemented in clinical practice, because the manual definition of the anatomies is time consuming.

In this study, we investigate an automatic anatomy segmentation method, with a view to overcoming the limitations of the bulk-density assignment method listed above. With this method, eight anatomies in the intracranial regions are defined and then assigned, i.e., body, air, eyeball, lens, cavity, ventricle, brainstem, and bone. These generated synthetic CT images are compared with water-equivalent and original CT images. We believe that the analysis of dose differences for evaluating these several approaches is not the proper method. This could be problematic because dose differences may be affected by the variations in RTP, such as size, location, and shape of tumors, or numbers, directional angles, and intensity of radiation beams. In other words, proper image similarity evaluation should be performed for verifying the generation of synthetic CT images from MR images. Therefore, this study aimed to provide the proper methodology to evaluate the generation of synthetic CT images for a radiation-dose calculation based on MR images.

## Materials and methods

**Fig 1:**
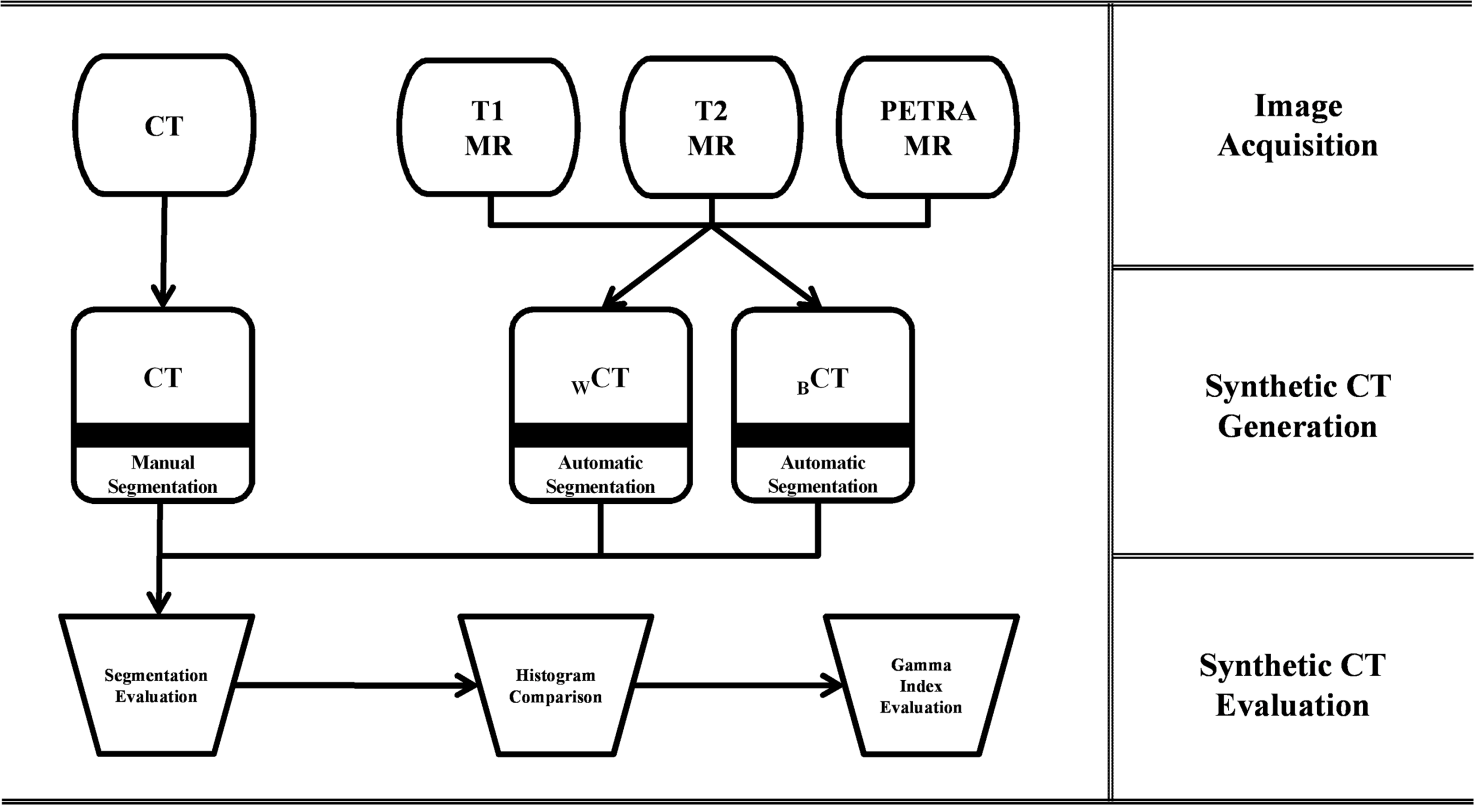
Schematic illustration of the procedures.

This study was performed through three major procedures—image acquisition, synthetic CT generation, and synthetic CT evaluation. The schematic illustration of this study is shown in Fig 1. Each procedure is detailed below.

### Image acquisition

Ethics approval for this study was obtained from the institutional review board (IRB). CT and MR data were collected for this study after IRB approval (IRB of the Catholic Medical Center, reference number: CIRB-00117_1–010). The IRB approved the exemption of this study from obtaining written informed consent because of its retrospective nature. CT images for the purpose of radiation treatment of 20 brain cancer patients were randomly selected. Their MR images acquired on the same day for the delineation of brain tumors were retrospectively collected. All the CT images were obtained using a LightSpeed RT 16 CT scanner (GE Medical Systems, Waukesha, WI), with the following CT scanning conditions—slice thickness 2.5 mm, peak voltage 120 kVp, current 433 mA (Auto), pitch 1.375, and display field of view 30 cm. All the MR images were obtained using a Skyra 3T MR scanner (Siemens Medical Systems, Erlangen, Germany). T1-weighted, T2-weighted, and pointwise encoding time reduction with radial acquisition (PETRA) MR images were acquired. The MR scanning conditions were as follows: T1-weighted MR images—echo time (TE) 2.5 ms, repetition time (TR) 250 ms, and flip angle (FA) 70°; T2-weighted MR images—TE 100 ms, TR 6310 ms, and FA 150°; PETRA MR images—TE 0.1 ms, TR 3.3 ms, and FA 6°. Other MR scanning conditions, such as slice thickness and display field of view, were the same as the CT scanning conditions.

### Synthetic CT generation

**Fig 2:**
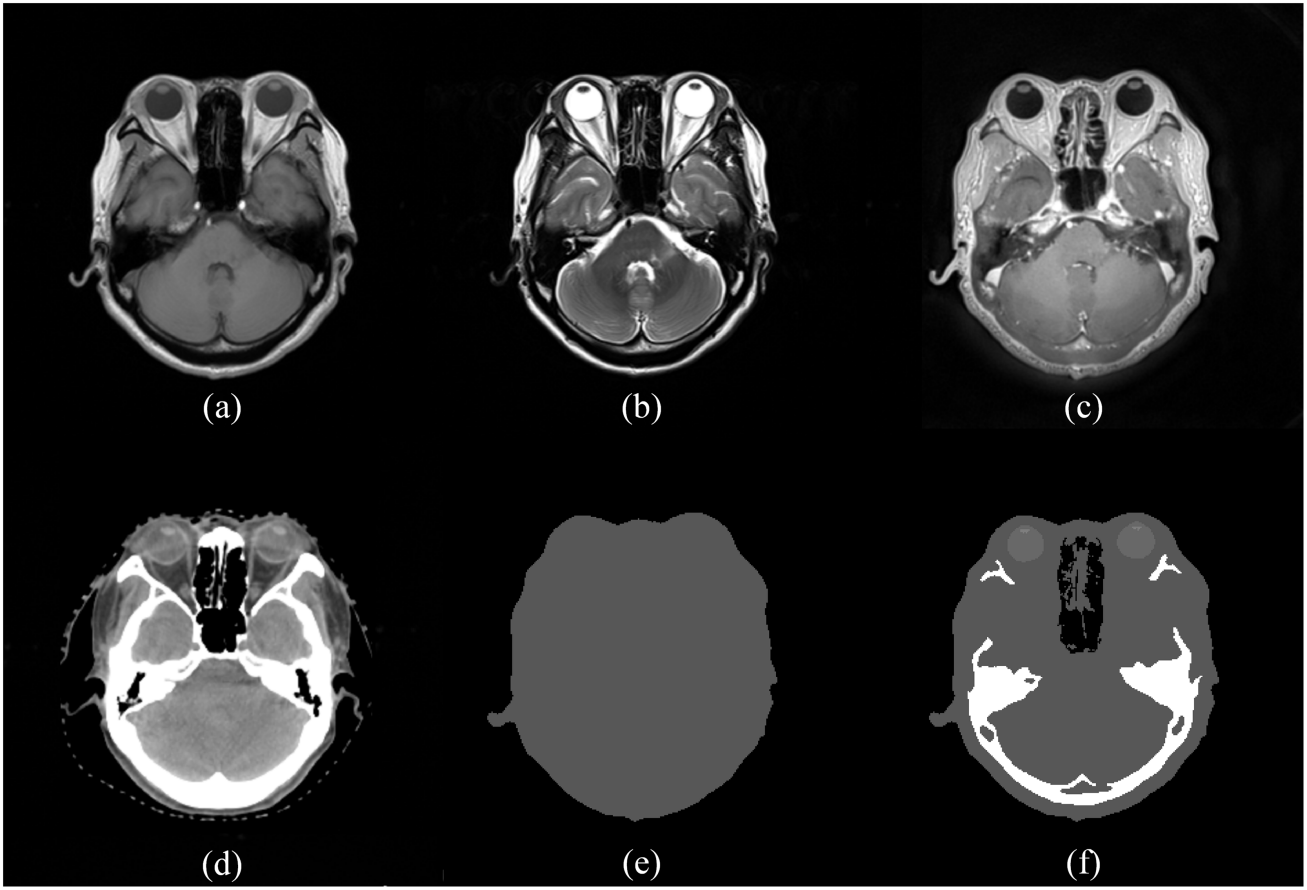
Acquired MR and CT image sets and generated synthetic CT image sets. (a) T1-weighted MR image. (b) T2-weighted MR image. (c) PETRA MR image. (d) Original CT image. (e) Water-equivalent synthetic CT (_W_CT). (f) Bulk-density-assigned CT image (_B_CT).

Two types of synthetic CT images were generated from MR images—water-equivalent CT images (_W_CT) and bulk-density-assigned CT images (_B_CT). By using MATLAB R2016a (Mathworks Inc., Natick, MA), a simple formula was applied to the three types of MR images, i.e. the T1-weighted, T2-weighted, and PETRA images for the automatic segmentation. An intensity-based method including thresholding and classification, and an atlas-based method including locational information are implemented for the automatic segmentation on the MR images. In the case of _W_CT, only two anatomies of the patient—body and air—were defined from MR images. Then, 0 and -1000 Hounsfield unit (HU) values were assigned to all internal and external areas, respectively. On the other hand, eight anatomies of the brain region of patients were segmented for _B_CT generation from MR images. Subsequently, each anatomy was individually assigned a defined HU value. Specifically, the air, bone, body, cavity, eyeball, lens, ventricle, and brainstem anatomies were assigned HU values of -1000, 1000, 0, -1000, 300, 300, 15, and -50, respectively. The obtained and generated CT image sets are shown in Fig 2.

### Synthetic CT evaluation

To evaluate the resulting _W_CT and _B_CT images, an affine and rigid registration procedure with the original CT images was performed. Eight anatomies, identical to those automatically segmented on MR images, were manually delineated on the original CT images to establish the ground truth. MIM Maestro 6.6.6 (MIM Software Inc., Cleveland, OH) was used for image registration and manual delineation. Subsequently, three distinct synthetic CT evaluation procedures were successively performed.

#### Performance of the automatic segmentation

The performance of the automatic segmentation method was quantitatively analyzed using the dice similarity coefficient (DSC), false negative dice (FND) coefficient, and false positive dice (FPD) coefficient [21]. The DSC, measuring the extent of spatial overlap between two binary images, is commonly used in evaluating the performance of segmentation. It ranges from zero to one; values of zero and one account for no overlap and perfect overlap, respectively. Moreover, larger FND and FPD account for under and over-segmentation, respectively. DSC, FND, and FPD are expressed as percentages in this study, and defined as:

$$DSC=\;\frac{2\left|A\cap G\right|}{\left|A\right|+\left|G\right|}\;\times \;100$$ (1)

$$FND=\;\frac{2\left|\bar{A}\cap G\right|}{\left|A\right|+\left|G\right|}\;\times \;100$$ (2)

$$FPD=\;\frac{2\left|A\cap \bar{G}\right|}{\left|A\right|+\left|G\right|}\;\times \;100$$ (3)

where A denotes the segmentation results and G denotes the ground truth. $\bar{A}$ and $\bar{G}$ are complements of the segmentation results and ground truth, respectively.

#### Histogram comparison

Histograms of the resulting _W_CT and _B_CT images were compared to those of the original CT images. Measuring the quantitative global image similarity between these resulting CT images and the original CT images is meaningful. In this study, four different histogram comparison standards were used for calculating the image similarity, viz. correlation, chi-square, intersection, and Bhattacharyya distance [22]. Microsoft Visual Studio 2015 (Microsoft Corp., Redmond, WA) and Open Source Computer Vision Library 3.0.0 (Intel Corp., Santa Clara, CA) were used. The mathematical equations of each method are given below.

$$D_{\text{correlation}}\left(H_{1},H_{2}\right)=\frac{\sum_{i}H_{1}^{'}\left(i\right)\cdot H_{2}^{'}\left(i\right)}{\sqrt{\sum_{i}H_{1}^{'2}\left(i\right)\cdot H_{2}^{'2}\left(i\right)}}$$ (4)

For correlation, a high value reveals a better match than a low value. A perfect match is one and a value of zero represents no correlation.

$$D_{\text{chi}-\text{square}}\left(H_{1},H_{2}\right)=\sum_{i}\frac{{\left(H_{1}\left(i\right)-H_{2}\left(i\right)\right)}^{2}}{H_{1}\left(i\right)+H_{2}\left(i\right)}$$ (5)

For chi-square, a low value represents a better match than a high value. A perfect match is zero and a mismatch is unbounded.

$$D_{\text{intersection}}\left(H_{1},H_{2}\right)=\sum_{i}\text{min}\left(H_{1}\left(i\right),H_{2}\left(i\right)\right)$$ (6)

For intersection, a high value reveals a better match than a low value. A perfect match is one and a total mismatch is zero.

$$D_{\text{Bhattacharyya}}\left(H_{1},H_{2}\right)=\sqrt{1-\underset{i}{\sum }\frac{\sqrt{H_{1}\left(i\right)\cdot H_{2}\left(i\right)}}{\sqrt{\sum_{i}H_{1}\left(i\right)\cdot \sum_{i}H_{2}\left(i\right)}}}$$ (7)

For Bhattacharyya distance, a low value represents a better match than a high value. A perfect match is zero and a total mismatch is one.

#### Gamma analysis

Gamma evaluation of the resulting CT and original CT images was performed. In radiation treatment, the gamma evaluation method is generally used to compare the predicted dose distribution and the acquired dose in terms of point doses [23]. In this study, the gamma evaluation measures local pixel value similarity of these images, whereas the histogram comparison measures the global pixel value similarity of these images. In other words, the gamma evaluation in this study was performed not to measure the differences of radiation doses in pixels, but the differences of CT numbers in pixels. Measuring the quantitative local image similarity between these resulting and original CT images is meaningful. The γ is calculated as:

$$\gamma =\sqrt{\frac{\Delta r^{2}}{\Delta d_{M}^{2}}+\frac{\Delta D^{2}}{\Delta D_{M}^{2}}}$$ (8)

where Δ*r* = |*r*_1_ − *r*_2_| denotes the distance between the reference and compared points, ΔD = *D*_2_(*r*_2_) − *D*_1_(*r*_1_) denotes the pixel difference at the position *r*_2_ relative to the reference pixel *D*_1_ in *r*_1_, Δd_*M*_ denotes the distance to agreement, and ΔD_*M*_ denotes the acceptance criteria. In this study, 3 mm and 3% criteria, 2 mm and 2% criteria, and 1 mm and 1% criteria were chosen. Gamma evaluations in this study were performed with the defined regions of interest as the minimum rectangular area covering the whole body and with the normalization between CT numbers of the synthetic CT and the original CT. To evaluate gamma values, OmniPro-I'mrt v1.7 (IBA Dosimetry, Schwarzenbruck, Germany) was used.

All the statistical analyses were performed using R v.3.3.2 (R Development Core Team, Vienna, Austria). In histogram comparison and gamma evaluation, the Welch two-sample t-test was used. A P-value of <0.05 was considered significant. Moreover, a power analysis with a power lever of 0.9 and significant level of 0.05 was performed, and the sample size was verified.

## Results

### Performance of the automatic segmentation

DSC, FND, and FPD of the eight anatomies are given in Table 1. The highest DSC value was 99.34 for air segmentation, and the lowest DSC value was 73.50 for bone segmentation. Moreover, the standard deviation (SD) of DSC was the highest for bone segmentation. Bone segmentation showed the worst performance for the automatic segmentation in all cases. For lens segmentation, the second-highest SD value was measured, and relatively high FND and FPD values were measured. Furthermore, the cavity and bone were measured as over-segmented anatomies having higher FPD than FND values. Except for bone segmentation, the segmentation results of other anatomies were considered to be in good agreement with the ground truth.

**Table 1:** Quantitative evaluation of the performance of the automatic segmentation methods.

	DSC (%)		FND (%)		FPD (%)	
	Mean	SD	Mean	SD	Mean	SD
Body	98.80	0.28	0.69	0.72	1.70	0.90
Air	99.34	0.36	1.02	0.75	0.31	0.32
Eyeball	96.67	2.77	3.37	3.37	3.29	3.60
Lens	89.60	8.36	10.44	15.59	10.36	9.81
Brainstem	94.76	4.21	6.05	6.27	4.44	5.49
Ventricle	95.56	3.25	4.03	5.07	4.85	4.41
Cavity	88.13	5.74	8.21	9.88	15.53	12.68
Bone	73.50	12.98	17.07	19.41	35.93	25.11

*Abbreviations*: dice similarity coefficient (DSC), false negative dice (FND), false positive dice (FPD), and standard deviation (SD).

### Histogram comparison

Four types of histogram comparisons were done, which are tabulated in Table 2. The averaged improvement of chi-square was the highest, and that of correlation was the lowest. The highest differential value with a perfect match value was measured as 1.40 at the chi-square comparison (perfect match: 0) between _W_CT and the original CT. Moreover, the lowest differential value with a perfect match value was measured as 0.06 at the correlation comparison (perfect match: 1) between _B_CT and the original CT. The measured histogram comparison results of _B_CT were better than those of _W_CT in all cases. All measured results were statistically significant (P < 0.001).

**Table 2:** Quantitative evaluation of the histogram comparison.

	Histogram comparison							
	Correlation		Chi-square		Intersection		Bhattacharyya	
	Mean	SD	Mean	SD	Mean	SD	Mean	SD
CT vs. _W_CT	0.90	0.02	1.40	0.58	0.73	0.05	0.44	0.02
CT vs. _B_CT	0.94	0.01	0.80	0.30	0.81	0.03	0.29	0.02
Improvement	0.05	0.01	0.41	0.08	0.08	0.02	0.33	0.04
P-value	< 0.001		< 0.001		< 0.001		< 0.001	

*Abbreviations*: computed tomography (CT), water-equivalent CT images (_W_CT), bulk-density-assigned CT images (_B_CT), and standard deviation (SD).

### Gamma analysis

**Fig 3:**
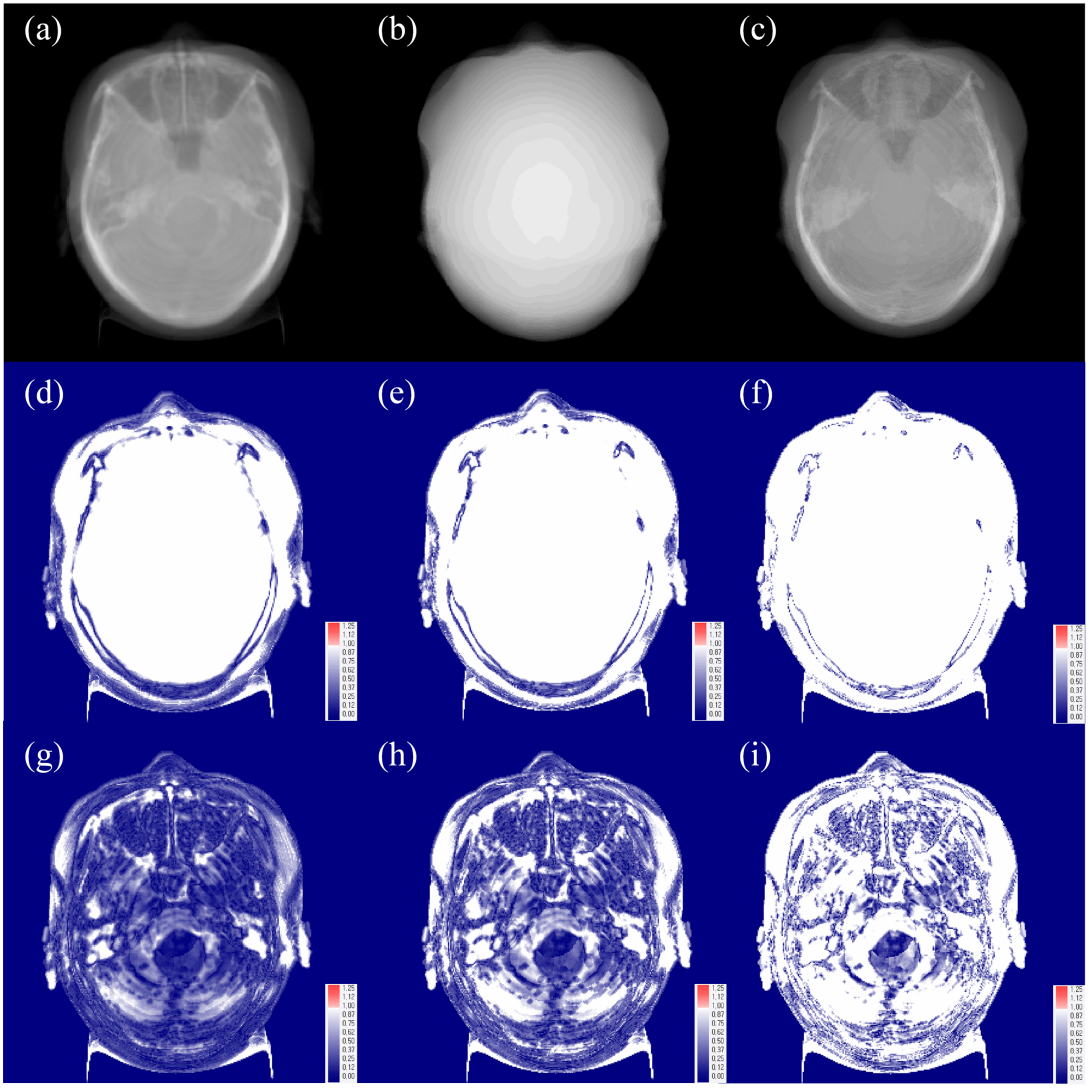
Gamma analysis between the original CT image and resultant synthetic CT images. (a) Original CT image. (b) Water-equivalent synthetic CT (_W_CT). (c) Bulk-density-assigned CT image (_B_CT). (d) Gamma analysis between the original CT and _W_CT with 3 mm/3% criteria. (e) Gamma analysis between the original CT and _W_CT with 2 mm/2% criteria. (f) Gamma analysis between the original CT and _W_CT with 1 mm/1% criteria. (g) Gamma analysis between the original CT and _B_CT with 3 mm/3% criteria. (h) Gamma analysis between the original CT and _B_CT with 2 mm/2% criteria. (i) Gamma analysis between the original CT and _B_CT with 1 mm/1% criteria.

The analyzed gamma values are shown in Fig 3 and Table 3. The averaged improvement of _B_CT compared to _W_CT was 33.25, 33.48, and 24.80 for the criteria of 3 mm/3%, 2 mm/2%, and 1 mm/1%, respectively. The measured gamma evaluation results of _B_CT were better than those of _W_CT in all 20 patient cases, and statistically significant (P<0.001).

**Table 3:** Quantitative evaluation of the gamma analysis.

	Gamma analysis					
	3 mm/3%		2 mm/2%		1 mm/1%	
	Mean	SD	Mean	SD	Mean	SD
CT vs. _W_CT	64.93	7.08	59.14	6.39	52.54	7.05
CT vs. _B_CT	87.63	9.39	78.86	9.69	65.29	8.87
Improvement	35.25	9.59	33.48	10.80	24.80	12.97
P-value	< 0.001		< 0.001		< 0.001	

*Abbreviations*: computed tomography (CT), water-equivalent CT images (_W_CT), bulk-density-assigned CT images (_B_CT), and standard deviation (SD).

## Discussion

In modern radiotherapy, multimodal clinical image acquisition techniques such as MRI and PET are generally utilized for accurate delineation of target volumes. However, these images do not provide electron-density information that enables radiation-dose calculation. Therefore, RTP systems based only on MRI have been extensively investigated, and several methods have been introduced for radiation-dose calculations based on MRI alone.

In addition, several authors proved that the bulk-density assignment method does not significantly compromise radiation-dose calculations. For example, Stanescu et al. manually segmented the brain, bone, and scalp on the MR images of eight patients, and assigned the corresponding HU values. These authors reported that the resultant dose difference was within 1% (compared to the dose-to-volume histogram (DVH) of the CT image-based dose calculation) [20]. Moreover, Saito et al. compared full-resolution CT and bulk-density-assigned CT for 70 lung cancer patients. They manually segmented air, lung, fat, soft tissue, and bone, and assigned HU values to each of them. They reported that normal the tissue DVH agreement was better than 2% in the dose and the planning target volume DVH was better than 3% in the dose [24]. Further, Jonsson et al. manually segmented the normal tissue, bone, lung, and air cavities in the MR images of 40 patients, and assigned HU values. These researchers reported a maximum dose difference of 1.6% [25]. To evaluate the generated CT image through a comparison with the corresponding MR image, Johansson et al. analyzed the deviation of the substitute CT from the MRI. These authors reported that the errors were large in both the high and low-density regions, and at the tissue interfaces [26].

In this study, we generated 20 brain cancer patients’ synthetic CT through the bulk-density assignment methods. Eight anatomies, including body, air, eyeball, lens, cavity, ventricle, brainstem, and bone, were automatically segmented with this method. In general, a DSC greater than 70 indicates excellent agreement [27]. All the measured averaged DSCs of all eight anatomies were over 70 as shown in Table 1. In this study, bone segmentation showed the worst performance for automatic segmentation as 73.50 DSC, and showed the over-segmentation tendency as 35.93 FPD. This may be a reason for the differences in pixel values between the bone area and abutted ones being significantly lower on all MR image sequences than other anatomies. In this study, according to the higher FPD of the bone and cavity, over-segmentation tends to exist when anatomies have low pixel values in all MR image sequences. For instance, the ventricle has low pixel values and high pixel values in T1 and T2-weighted MR images, respectively, unlike the bone and cavity. Furthermore, the second-highest SD value was measured for lens segmentation, and high FND and FPD values were measured. This may be caused by lenses having small volumes.

Histogram comparison and gamma analysis were performed to evaluate the image similarity between synthetic CT and the original CT images. The highest differential value with a perfect match value was measured as 1.40 for the chi-square comparison between _W_CT and the original CT. This may be caused by chi-square having an unbounded value unlike the others. Compared to the values of the perfect match case in the histogram comparison, excellent histogram matches were observed, as given in Table 2. The evaluated gamma measurement results of _B_CT were better than those of _W_CT in all 20 patients’ cases with all criteria. Even though the results of the gamma index evaluation are considered to have low passing rates compared to the conventional gamma index analysis for radiation dose differences, the improvement in the results between _W_CT and _B_CT validates this gamma-index analysis methodology. According to the results of the histogram comparison and gamma analysis, _B_CT is significantly better than _W_CT in terms of both local and global pixel value comparisons.

This study was conducted only on the intracranial regions. Moreover, the immobilization device and flat table used in the original CT scans were not utilized because this study was retrospectively implemented. Geometrical distortions of the MR images were not considered. However, the differences between the original CT and obtained MR images were minimal because this study was implemented on the intracranial regions. Furthermore, this study intended to provide the proper methodology for evaluating the generation of synthetic CT images for a radiation-dose calculation based on MR images. Better image similarity between _B_CT and the original CT could be possible when those limitations are solved with MR simulation for radiation treatment.

## Conclusion

In this study, the automatic bulk-density assignment method was successfully implemented for eight defined anatomies in the intracranial region. The image similarity results were properly evaluated, and showed that _B_CT has superior results compared to _W_CT for all measurements. Consequently, more accurate radiation treatment for the intracranial regions can be expected when the proper image similarity evaluation introduced in this study is performed.
